# Label-Free and Homogeneous Electrochemical Biosensor for Flap Endonuclease 1 Based on the Target-Triggered Difference in Electrostatic Interaction between Molecular Indicators and Electrode Surface

**DOI:** 10.3390/bios12070528

**Published:** 2022-07-15

**Authors:** Jianping Zheng, Xiaolin Xu, Hanning Zhu, Zhipeng Pan, Xianghui Li, Fang Luo, Zhenyu Lin

**Affiliations:** 1 Department of Oncology, Shengli Clinical Medical College, Fujian Medical University, Fujian Provincial Hospital, Fuzhou 350001, China; fjslzjp@sina.com; 2 Department of Clinical Laboratory, School of Medical Technology and Engineering, Fujian Medical University, Fuzhou 350004, China; 1696219821@fjmu.edu.cn (X.X.); zhuhanning95@163.com (H.Z.); panzp0109@163.com (Z.P.); 3 Ministry of Education Key Laboratory for Analysis Science of Food Safety and Biology, Fujian Provincial Key Laboratory of Analysis and Detection for Food Safety, College of Chemistry, Fuzhou University, Fuzhou 350116, China; luofang@fzu.edu.cn

**Keywords:** homogeneous, electrochemical biosensor, label-free, hyperbranched rolling circle amplification, flap endonuclease 1

## Abstract

Target-induced differences in the electrostatic interactions between methylene blue (MB) and indium tin oxide (ITO) electrode surface was firstly employed to develop a homogeneous electrochemical biosensor for flap endonuclease 1 (FEN1) detection. In the absence of FEN1, the positively charged methylene blue (MB) is free in the solution and can diffuse onto the negatively charged ITO electrode surface easily, resulting in an obvious electrochemical signal. Conversely, with the presence of FEN1, a 5′-flap is cleaved from the well-designed flapped dumbbell DNA probe (FDP). The remained DNA fragment forms a closed dumbbell DNA probe to trigger hyperbranched rolling circle amplification (HRCA) reaction, generating plentiful dsDNA sequences. A large amount of MB could be inserted into the produced dsDNA sequences to form MB-dsDNA complexes, which contain a large number of negative charges. Due to the strong electrostatic repulsion between MB-dsDNA complexes and the ITO electrode surface, a significant signal drop occurs. The signal change (Δ*Current*) shows a linear relationship with the logarithm of FEN1 concentration from 0.04 to 80.0 U/L with a low detection limit of 0.003 U/L (S/N = 3). This study provides a label-free and homogeneous electrochemical platform for evaluating FEN1 activity.

## 1. Introduction

Flap endonuclease 1 (FEN1) exhibits multiple values in the early diagnosis [1,2], targeting therapy [3,4,5,6], and prognostic monitoring [7,8] of various cancers. Traditional assays, including western blot, reverse transcription-polymerase chain reaction (RT-PCR), and enzyme-linked immunosorbent assay (ELISA) [1,3,4] had already been utilized to detect FEN1. Several novel strategies were also designed for FEN1 detection [9,10,11,12,13,14,15]. For instance, Zhang et al. [9] proposed a DNA-based fluorescent biosensor to evaluate FEN1 activity in living cells. Our group developed an electrochemiluminescence (ECL) biosensor for FEN1 via combining branched hybridization chain reaction (BHCR) amplification, ultrafiltration separation, and ECL detection [16]. Although these methods can realize FEN1 detection with high accuracy, it is still desirable to explore novel analytical methods with increased performance and decreased cost.

Electrochemical analysis combining the merits of low cost, simple operation, high sensitivity, and fast response has been extensively used in biological and biomedical applications [17,18]. However, most electrochemical sensors require the laborious and time-consuming immobilization of recognition probes on the electrode surface, labeling of reporter molecules first. Additionally, target recognition takes place on the interface between solution and electrode, which lowers the reaction efficiency and recognition rates because of the steric hindrance. Immobilization-free homogeneous electrochemical methods have been developed on the basis of the difference in the electrostatic interaction between DNA sequences and the negatively charged indium tin oxide (ITO) electrode can address these concerns well [19,20,21]. Methylene Blue (MB), as a widely used electrochemical indicator, is a positively charged organic dye that can be inserted into double-stranded DNA (dsDNA) through π–π stacking interactions [22]. Taking advantage of its special interaction with dsDNA, MB has been adopted to design an enzyme-free and label-free homogeneous electrochemical miRNA biosensor via the difference in electrostatic repulsion between MB-intercalated dsDNA and ITO electrode [23]. However, as far as we know, this strategy had not been applied to detect FEN1 activity.

Hyperbranched rolling circle amplification (HRCA) [24] evolved from rolling circle amplification (RCA) and is a simple and convenient method with much higher isothermal amplification efficiency (10^9^-fold) than that of RCA [25]. This can produce a large number of dsDNAs with high efficiency. In this study, a label-free and homogeneous electrochemical biosensor has been designed for monitoring FEN1 activity by combining the well-designed flapped dumbbell DNA probe (FDP), target-induced electrostatic interactions between MB molecules, and negatively charged dsDNA strands, and the excellent HRCA technology. This homogeneous biosensor can avoid the complicated electrode modification, high cost of labeling, and steric hindrance during the HRCA, which guarantees that the detection process will be simpler and faster, while achieving low costs and good reproducibility. Additionally, the proposed biosensor was used to measure the FEN1 levels in clinical samples with satisfied performance, and therefore, could serve as a potent platform for monitoring FEN1 activity in clinical diagnosis.

## 2. Experimental Section

### 2.1. Reagents and Oligonucleotides

The lysates of AGS and HaCaT cells were prepared and stored at −20 °C for the following assays (see the details in the Supplementary Information (SI)). All the other reagents and chemicals were analytical grade. Ultrapure water with a resistance of 18.2 MΩ∙cm was adopted in the whole experiment. The designed oligonucleotides were synthesized by Shanghai Sangon Biotechnology Co., Ltd. (Shanghai, China) with the following sequences:

FDP: 5′-TTAACGACCATTCAAACGCACTGATGGTTGCCAACCACAAACGGCA A-3’

P1: 5′-CAGTGCGTT-3’

P2: 5′-ACCACAAAC-3’

The other materials and reagents employed in this experiment are listed in the SI.

### 2.2. Apparatus

Differential pulse voltammetry (DPV) signals were measured by a CHI660a electrochemical workstation (Chenhua Instruments, Shanghai, China). ITO electrode served as a working electrode, and platinum wires were utilized as the reference electrode and the auxiliary electrode, respectively. ITO electrodes were bought from Huanan Xiangcheng Technology Co., Ltd. (Shenzhen, China). Before DPV signals were acquired, the surface of the ITO electrode was firstly decorated with negative charges by sequentially sonicating it in Alconox solution (20 g/L), 2-propanol, and ultrapure water for 15 min. The ITO electrode was then inserted vertically into the Nafion solution (0.5 mg/mL) for 10 s and then removed and dried to quickly prepare a negatively charged electrode. The operating area of the ITO electrode was set to 3 mm × 3 mm.

In total, 12% polyacrylamide gel electrophoresis was employed to notarize the occurrence of each course in the HRCA reaction. Gel electrophoresis imager (JS-2012) was obtained from Shanghai Peiqing Technology Co., Ltd. (Shanghai, China). The test was performed in 0.5× triborate-EDTA (TBE) buffer (pH 8.4) under a constant voltage of 90 V for 60 min at room temperature. SYBR Green I (20×) was added to the amplification product and incubated at room temperature in the dark for 15 min. Then, the fluorescence signals were detected by an F-4600 Fluorescence spectrofluorometer purchased from Hitachi high-technologies corporation (Tokyo, Japan) in the range of 500~650 nm with the excitation wavelength setting to 488 nm.

### 2.3. Process of FEN1 Detection

The test sample (10 μL), FDP (10 nM), and reaction buffer (3 mM MgSO_4_, 15 mM (NH_4_)_2_SO_4_, 30 mM Tris-HCl, and 15 mM KCl, pH 8.8) of 30 μL were mixed and incubated at 37 °C for 70 min. Afterward, T4 DNA ligase (2 μL, 5 U/μL) was added to the system and incubated at 22 °C for 70 min with a corresponding 1× reaction buffer. Then, the superfluous ssDNA and dsDNA probes were removed by Exo I (10 U) and Exo III (20 U) at 37 °C for 1 h and further inactivated at 80 °C for 15 min. Subsequently, primers (P1, P2, 1 μM), dNTPs (0.4 mM), Bst DNA polymerase (80 U/mL), and 1× reaction buffer were mixed and amplified for 100 min at 63 °C. Finally, MB (40 μM) was added to the 200 μL total reaction system, and the current signal was detected.

## 3. Results and Discussion

### 3.1. Principle of the Proposed Biosensor for FEN1

**Scheme 1:**
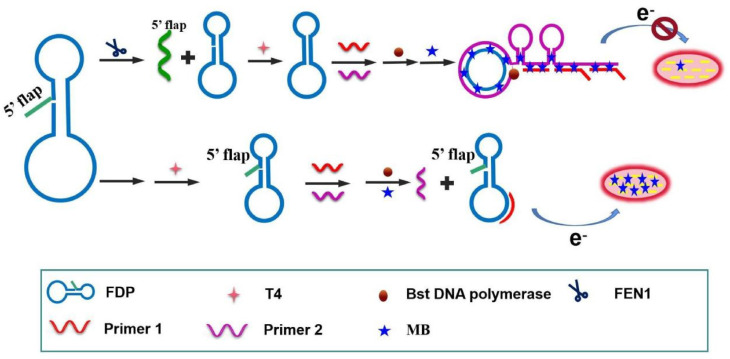
Scheme of the homogeneous electrochemical biosensor for FEN1 detection based on the difference in electrostatic interaction.

Scheme 1 clearly exhibits the principle of the designed strategy for FEN1 detection. Firstly, FDP with a 5′-flap that can be identified and cleaved by FEN1 was rationally designed. Free MB molecules have positive charges, and the ITO electrode carries negative charges. Thus, MB can diffuse freely onto ITO to generate high electrochemical signals. In the presence of FEN1, 5′-flap is separated from FDP and leaves an exposed 5′ phosphate group. Due to the stabilization of DNA scaffold the 5′ phosphate approaches the 3′-flap to form a nick site that is ligatable by T4 DNA ligase. After ligation, a closed dumbbell DNA probe (C-DNA) with a circular conformation is formed. Then, only C-DNA probes remained by adding Exo I and III to the solution. Afterward, the HRCA mixture, including primers, dNTPs, Bst DNA polymerase, and MB, was added to the above solution. After initiating the HRCA reaction, a large amount of dsDNA was produced, and abundant MB can be inserted into these dsDNA products to form MB-dsDNA complexes. The resulting MB-dsDNA complexes carry negative charges. Hence, a significantly reduced electrochemical signal was detected. Therefore, the recorded electrochemical signal of this system is related to the concentration of the FEN1 target, resulting in a label-free and immobilization-free electrochemical biosensor for detecting the FEN1 activity. In contrast, without FEN1, the 5′-flap could not be cleaved and the ligation would not occur. So, the current signal of the solution hardly changed.

### 3.2. Feasibility Test

**Figure 1:**
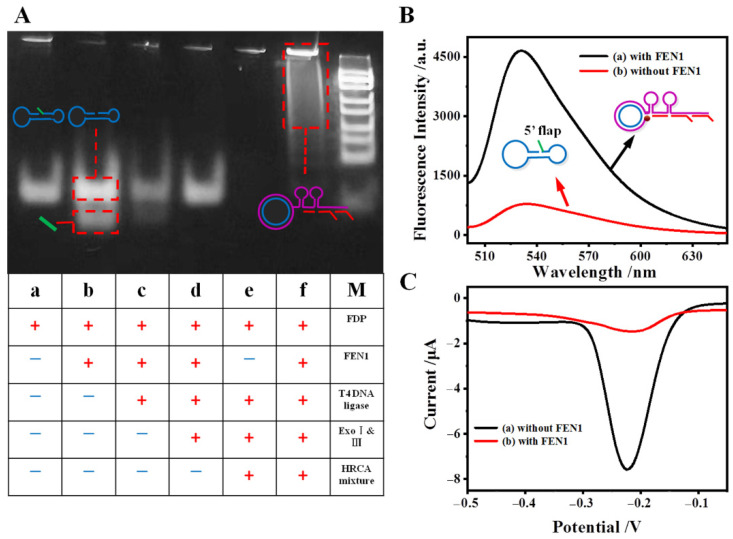
Feasibility study. (**A**) Polyacrylamide gel electrophoresis under different conditions: (a) FDP, (b) FDP + FEN1, (c) FDP + FEN1 + T4, (d) FDP + FEN1 + T4 + Exo I and III, (e) FDP + T4 + Exo Ⅰand Ⅲ + HRCA mixture, (f) FDP + FEN1 + T4 + Exo I and III + HRCA mixture, (M) marker. (**B**) Fluorescence spectra at present and absence of FEN1: (a) with FEN1, (b) without FEN1. (**C**) The DPV responses with and without target: a) without FEN1; (b) with FEN1. The concentrations of FEN1 and FDP are 80 U/L and 5 nM, respectively.

First of all, polyacrylamide gel electrophoresis, as the gold standard to analyze nucleic acids for length, bend, and flexibility was used to ensure the feasibility of the HRCA strategy. As displayed in Figure 1A, seven lanes were observed in the image of polyacrylamide gel electrophoresis. Lane a with a single bright band represents FDP in the monomeric and uniform state. Upon the addition of FEN1, the 5′-flap of FDP can be cleaved by the FEN1 target, forming a dumbbell DNA and a short DNA. Thus, lane b contains two obvious bands, in which the lower band corresponds to the dissociated flap sequences and the upper band denotes the closed dumbbell-shaped DNA. This lane clearly evidences the specific recognition ability of FEN1 to cleave the 5′-flap structure on FDP. Similar to lane b, the mixture of FDP, FEN1, T4 DNA ligase, and the necessary buffer also generated two bands in lane c. Different from lane c, only one bright band is left in lane d after adding Exo I and III, with the same position as the uppermost band in lane c. This verifies the above conjecture that a closed circular dumbbell-shaped DNA is indeed generated under the action of T4 ligase. Finally, with the addition of the HRCA mixture, a band with tailing appears in lane f, which corresponds to the products of the HRCA reaction. Conversely, without FEN1 targets, no 5′-flap can be cleaved from FDP, and thus the HRCA cannot be induced to occur. Therefore, no obvious bands emerged in lane e. All in all, these results clearly revealed that FEN1 indeed could cleave the 5′-flap of FDP to initiate the subsequent HRCA process.

In addition, fluorescence spectra have been also utilized to verify the proposed sensing scheme (Figure 1B). SYBR Green I could be embedded into the holes of dsDNA, yielding a strong fluorescent intensity at 530 nm. When FEN1 is added, an obvious fluorescence emission could be recorded at 530 nm (curve a), indicating that plentiful dsDNAs were produced. In contrast, in the absence of FEN1, very low fluorescence intensity was recorded (curve b) because the FDP also has a certain amount of complementary structure. It is demonstrated that the FDP, T4 DNA ligase, and HRCA mixture are not capable to induce HRCA to generate a large amount of dsDNA.

The feasibility of this sensor can be also verified by the change in the zeta potential of the studied system before and after the reaction. As shown in Figure S1, a negative potential of −40.99 mV was observed for the product of the sample with FEN1, which can be attributed to the negatively charged phosphate on the amount of dsDNA molecular skeleton. Concurrently, a negative potential of −8.06 mV was obtained for the product of the sample without FEN1, indicating that no amplification reaction occurred in this system. After incubating with positively charged MB, the zeta potential of the experimental group changed to −26.65 mV, while the zeta potential of the control group turned into 2.62 mV. This fact confirms the completion of the amplification reaction and successful embedding of MB.

Furthermore, DPV responses were tested to examine this proposed sensing scheme. As shown in Figure 1C, the DPV signal is significant in the absence of FEN1 (curve a). This is because the amplification reaction cannot be initiated, which will not affect the MB in the solution to freely diffuse to the electrode surface. When the FEN1 targets are present, an obvious decrease in DPV signal can be found (curve b) because the HRCA reaction generates a great amount of dsDNA and the DPV signal reporter MB molecules are mainly embedded into the resulting dsDNA sequences. Thus, only a fraction of remaining MB molecules can diffuse onto the ITO electrode surface to yield a much weaker DPV signal.

### 3.3. Optimization of the Experimental Conditions

**Figure 2:**
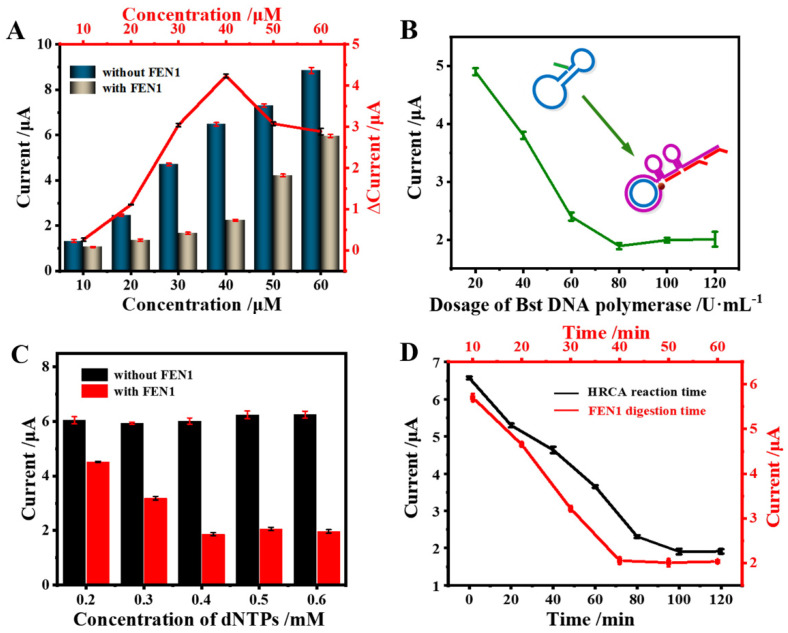
Condition optimization: (**A**) concentration of MB; (**B**) dosage of Bst DNA polymerase; (**C**) concentration of dNTPs; (**D**) HRCA reaction time and FEN1 digestion time. The concentrations of FEN1 and FDP are 0.5 U/L and 5 nM, respectively.

To reveal the best performance of this homogeneous electrochemical sensor, the influence of FEN1 digestion time, HRCA reaction, and the concentration of MB on the final readout have been investigated. Amongst these, the concentration of MB is a vital factor for the performance of biosensors because an appropriate concentration of MB could assure a satisfactory detection range and low background response. Firstly, the HRCA products were treated with different concentrations of MB for 1 h. From Figure 2A, it can be seen that the DPV responses of both the blank sample and the one with FEN1 of 0.8 U/L gradually increase with the increasing MB concentration. However, Δ*Current* values, namely the difference in current in absence and presence of FEN1, progressively increase and subsequently decrease slightly when the concentration of MB reaches 40 μM. Thereby, the optimal MB concentration is settled as 40 μM.

The dosage of Bst DNA polymerase can directly affect the efficiency of HRCA. Figure 2B shows that, along with the raising dosage of Bst DNA polymerase, the DPV peak current sharply declines until 80 U/mL. After that, the DPV signals hardly change anymore, which reveals that 80 U/mL of Bst DNA polymerase is suitable for the HRCA reaction. For HRCA reaction, the concentration of dNTPs is also pivotal. As shown in Figure 2C, when the number of used dNTPs is more than 0.4 mM, the DPV current of the sample stops decreasing, indicating that 0.4 mM is the minimum amount of dNTPs needed for the HRCA reaction.

Subsequently, different digestion time intervals and reaction times were evaluated. As presented in Figure 2D, the current response firstly drops, and then reaches a plateau after 70 min of digestion, suggesting that 70 min is long enough for completely cleaving the flap of FDP. It is also observed from Figure 2D that, before reaching the lowest point at 100 min, the current is almost proportional to the HRCA reaction time. The current hardly changes after 100 min of the HRCA reaction, which implied that 100 min is enough for the amplification reaction. Such tests clearly demonstrate that the best digestion duration is 70 min, and the suitable HRCA reaction time is 100 min. Hence, these optimized conditions have been used in the following assays.

### 3.4. Performance of the Developed Homogeneous Electrochemical Biosensor

**Figure 3:**
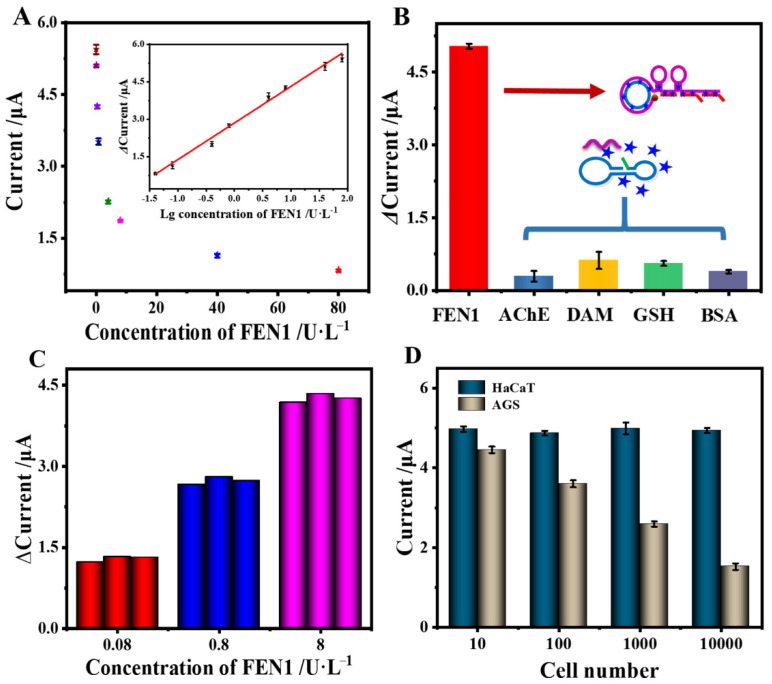
(**A**) Relationship between DPV peak current and FEN1 concentration. Insert: Calibration curve between Δ*Current* and the logarithm of the target FEN1 concentration. (**B**) Selectivity of the proposed homogeneous electrochemical biosensor in the presence of 8 U/L FEN1 and other interferences. (**C**) Reproducibility of the proposed electrochemical biosensor. (**D**) Test in the cell lysates of HaCaT and AGS cells by the proposed sensor. The error bars show the standard deviation of three replicate determinations.

Under the optimal conditions, the analytical performance of this homogeneous electrochemical biosensor was evaluated by testing a series of samples containing different concentrations of FEN1. From Figure 3A, it is observed that, with the raising target concentration, the current intensity of the testing systems decreases synchronously. The inset of Figure 3A showed that there is a linear relationship between the Δ*Current* and the logarithmic concentration of FEN1 from 0.04 to 80.0 U/L. The fitted linear equation is as follows:

Δ*Current* = 1.47 lg*C*_FEN1_ + 2.85  *R^2^* = 0.996

where *C* represents the concentration of FEN1 (U/L), *R* is the meaning of the correlation linear coefficient, and Δ*Current* is expressed in units of μA. The limit of detection (LOD) of this biosensor is estimated to be 0.003 U/L (S/N = 3). As compared with the reported sensors for FEN1 detection [12,13], this designed biosensor has a wide dynamic range, higher sensitivity, and lower LOD since the HRCA reaction has greatly amplified the detecting signals. That is to say, this designed homogeneous electrochemical biosensor has a desirable linear range and high sensitivity, showing great potential in assessing FEN1 activity.

Satisfactory selectivity and anti-interference are prerequisites for the practical applicability of a sensing platform. The selectivity of this homogeneous electrochemical biosensor was verified by choosing acetylcholinesterase (AChE), Dam methyltransferase (DAM), glutathione (GSH), and bovine serum albumin (BSA) as interferences. Here, the interferences concentration was set to 100 U/L, BSA was set to 50 μg/mL, and FEN1 concentration was set to 8 U/L. None of these interferences can cleave 5′-flap from the FDP substrate to generate a dumbbell-shaped padlock probe, and thus, no HRCA can be initiated. As a result, as shown in Figure 3B, the DPV signal changes (Δ*Current*) after adding these interferences are much smaller than significant signal changes upon the addition of the FEN1. These results suggest that this designed biosensor has good specificity for FEN1 detection and prominent selectivity towards its analogs.

The reproducibility of this proposed electrochemical biosensor was evaluated by repeated assays three times at 0.08 U/L, 0.8 U/L, and 8 U/L, respectively (see Figure 3C). The root square deviation (RSD) of each group (*n* = 3) at the equal concentration is 4.3%, 2.6%, and 1.9%, respectively, indicating that this proposed biosensor has good reproducibility. In addition, stability is also an important indicator to confirm the excellent performance of biosensors. Five treated ITO electrodes were used to detect the same sample (2.0 U/L FEN1) to obtain the stability curves, as shown in Figure S2. The RSD of current intensity is 2.6% (*n* = 5), meaning that this electrochemical biosensor has satisfactory stability. These test results verify the superior performance of this proposed electrochemical sensing platform.

### 3.5. Application of Biosensor to Detect FEN1 in Practical Samples

To assess the capability of the designed biosensor in practical application, this electrochemical biosensor was further applied to evaluate the FEN1 levels in cell lysates. Two cell lines, i.e., AGS (the FEN1 level positively correlated with the progression of gastric cancer) and HaCaT (the FEN1 level barely changed) were selected. As shown in Figure 3D, a significant current signal drop has been observed after adding the AGS cell lysates in the range of 10~10^4^ cells, while the current scarcely decreases upon adding the HaCaT cell lysates with the equivalent cell number. This indicates that the proposed homogeneous biosensor has a satisfactory performance in the practical evaluation of FEN1 activity.

## 4. Conclusions

In this study, a label-free homogeneous electrochemical biosensor with ultra-high sensitivity and selectivity was proposed for FEN1 activity detection based on the HRCA technology. The well-designed FDP with 5′-flaps can be cleaved by FEN1 to form the dumbbell DNA probes, which can trigger HRCA reactions to generate dsDNA sequences. The resulting dsDNA could hinder the MB indicators to diffuse onto the IPO electrode surface, which leads to a decrease in electrochemical signals. The signal change had a direct relationship with the FEN1 concentration. Moreover, this biosensor is capable of quantifying the FEN1 activity in cell lysates with satisfactory results, indicating that this proposed electrochemical strategy has the potential to serve as a new method for FEN1 clinical assay.

## Supplementary Materials

The following supporting information can be downloaded at: https://www.mdpi.com/article/10.3390/bios12070528/s1, The details of materials and reagents, cell culture and protein extraction; Figure S1: Zeta potential characterization of this sensor; Figure S2: Stability of the proposed biosensor.
