# Supplementary material for: Label-Free and Homogeneous Electrochemical Biosensor for Flap Endonuclease 1 Based on the Target-Triggered Difference in Electrostatic Interaction between Molecular Indicators and Electrode Surface
Source: Biosensors (Basel). 2022 Jul 15;12(7):528. doi: 10.3390/bios12070528 (PMC9313405; doi:10.3390/bios12070528)
Supplement: Supplementary file 1 [file biosensors-12-00528-s001.zip › biosensors-1817275-supplementary.pdf]

Supplementary Information

Label-Free and Homogeneous Electrochemical Biosensor for Flap Endonuclease 1 Based on the Target-Triggered Difference in Electrostatic Interaction between Molecular Indicators and Electrode Surface

Jianping Zheng [1], Xiaolin Xu [2], Hanning Zhu [2], Zhipeng Pan [2], Xianghui Li [2,*], Fang Luo [3] and Zhenyu Lin [3,*]

[1] Department of Oncology, Shengli Clinical Medical College of Fujian Medical University, Fujian Provincial Hospital, Fuzhou 350001, China; fjslzjp@sina.com

[2] Department of Clinical Laboratory, School of Medical Technology and Engineering, Fujian Medical University, Fuzhou 350004, China; 1696219821@fjmu.edu.cn (X.X.); zhuhanning95@163.com (H.Z.); panzp0109@163.com (Z.P.)

[3] Ministry of Education Key Laboratory for Analysis Science of Food Safety and Biology, Fujian Provincial Key Laboratory of Analysis and Detection for Food Safety, College of Chemistry, Fuzhou University, Fuzhou 350116, China; luofang@fzu.edu.cn

* Correspondence: lixianghui1987@126.com (X.L.); zylin@fzu.edu.cn (Z.L.); Tel./Fax: +86-591-22866135

Table of Content

1. Experimental section

1.1. Materials and reagents.

1.2. Cell culture and protein extraction.

2. Supplementary figures

Figure S1. Zeta potential characterization of this sensor.

Figure S2. Stability of the proposed biosensor.

1. Experimental section

1.1. Materials and reagents.

T4 DNA Ligase, Bst DNA polymerase (large fragment) with corresponding buffer solutions, deoxynucleotide (dNTPs) were obtained from New England BioLabs (Beijing, China). FEN1 in conjunction with buffer solutions was bought from NEB (Beijing, China). Exonuclease I and III (Exo I and III) were purchased from Thermo Fisher Scientific (Shanghai, China). SYBR Green I (10,000×) was supplied by Xiamen Biovision Biotechnology Co., Ltd. (Xiamen, China). 20× PBS buffer was acquired from Aladdin Chemistry Co. (Shanghai, China). Methylene blue (MB) was sourced from Sinopharm Chemical Reagent Co., Ltd. (Shanghai, China).

1.2. Cell Culture and Protein Extraction.

Human immortalized epidermal cells (HaCaT) and human gastric adenocarcinoma cells (AGS) were cultured in DMEM supplemented with 10% fetal bovine serum (FBS), 100 U/mL penifcillin and 100 μg/mL streptomycin, in a humidified atmosphere with 5% CO_2 at 37 °C. The cellular extracts were harvested by RIPA according to the protocol. Finally, the obtained total protein extracts were diluted and stored at -20 °C before using for further FEN1 detection.

2. Supplementary figures

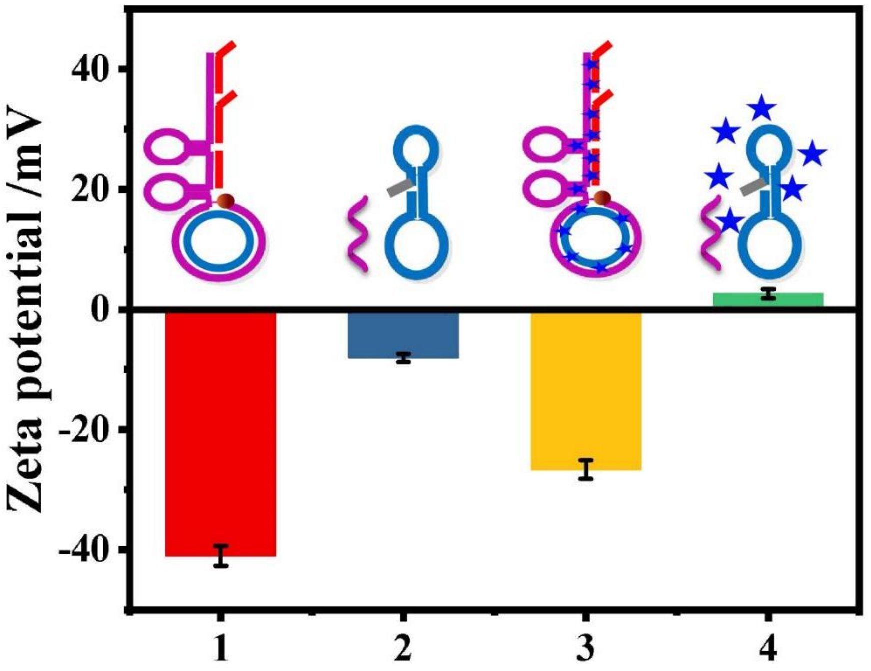

Figure S1. Zeta potential characterization of this biosensor.

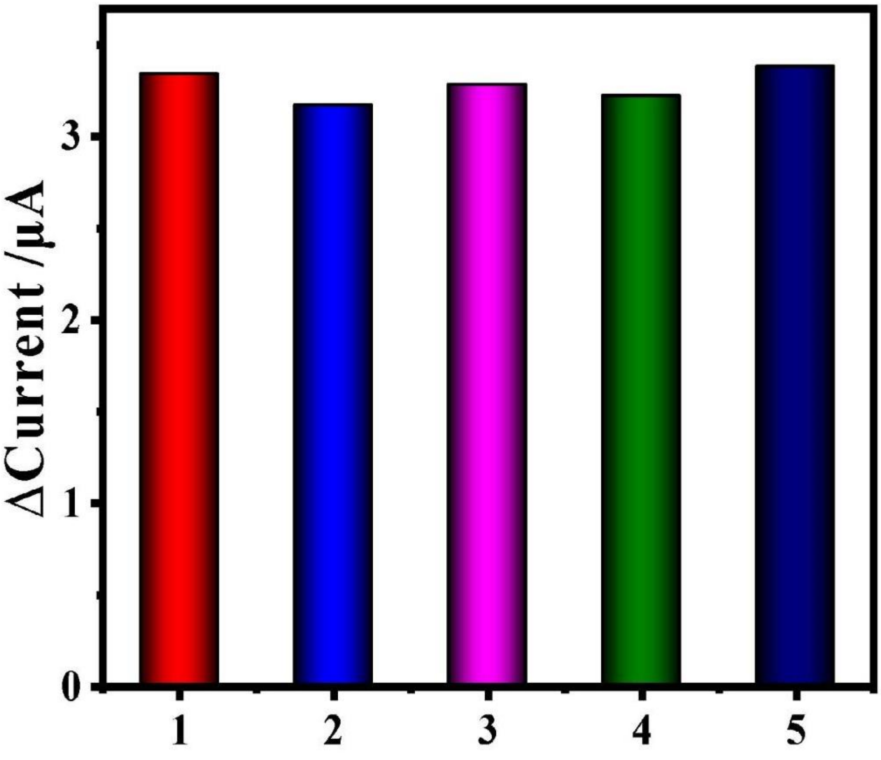

Figure S2. Stability of the proposed biosensor.
